# Structure, Optical and Magnetic Properties of Two Isomeric 2-Bromomethylpyridine Cu(II) Complexes [Cu(C_6_H_9_NBr)_2_(NO_3_)_2_] with Very Different Binding Motives

**DOI:** 10.3390/molecules28020731

**Published:** 2023-01-11

**Authors:** Fatma Garci, Hammouda Chebbi, Nahal Rouzbeh, Leonhard Rochels, Sabrina Disch, Alexander Haseloer, Sean S. Sebastian, Uwe Ruschewitz, Eric Tobechukwu Anthony, Axel Klein, Mohamed Faouzi Zid

**Affiliations:** 1 University of Tunis El Manar, Faculty of Sciences of Tunis Laboratory of Materials, Crystal Chemistry and Applied Thermodynamics, El Manar II, Tunis 2092, Tunisia; 2 University of Cologne, Faculty of Mathematics and Natural Sciences, Department of Chemistry, Institute for Inorganic Chemistry, Greinstrasse 6, D-50939 Köln, Germany; 3 University of Tunis, Preparatory Institute for Engineering Studies of Tunis, Jawaharlal Nehru Street, Montfleury, Tunis 1089, Tunisia; 4 University of Cologne, Faculty of Mathematics and Natural Sciences, Department of Chemistry, Institute for Physical Chemistry, Greinstrasse 4–6, D-50939 Köln, Germany

**Keywords:** copper(II), 2-bromomethylpyridine, stereoisomers, magnetization, EPR

## Abstract

Two isomeric 2-bromomethylpyridine Cu(II) complexes [Cu(C_6_H_9_NBr)_2_(NO_3_)_2_] with 2-bromo-5-methylpyridine (L^1^) and 2-bromo-4-methylpyridine (L^2^) were synthesized as air-stable blue materials in good yields. The crystal structures were different with [Cu(L^1^)_2_(NO_3_)_2_] (**CuL^1^**) crystallizing in the monoclinic space group *P*2_1_/c, while the 4-methyl derivative **CuL^2^** was solved and refined in triclinic *P*$\bar{1}$. The orientation of the Br substituents in the molecular structure (*anti* (**CuL^1^**) vs. *syn* (**CuL^2^**) conformations) and the geometry around Cu(II) in an overall 4 + 2 distorted coordination was very different with two secondary (axially elongated) Cu–O bonds on each side of the CuN_2_O_2_ basal plane in **CuL^1^** or both on one side in **CuL^2^**. The two Br substituents in **CuL^2^** come quite close to the Cu(II) centers and to each other (Br⋯Br ~3.7 Å). Regardless of these differences, the thermal behavior (TG/DTA) of both materials is very similar with decomposition starting at around 160 °C and CuO as the final product. In contrast to this, FT-IR and Raman frequencies are markedly different for the two isomers and the UV–vis absorption spectra in solution show marked differences in the π–π* absorptions at 263 (**CuL^2^**) or 270 (**CuL^1^**) nm and in the ligand-to-metal charge transfer bands at around 320 nm which are pronounced for **CuL^1^** with the higher symmetry at the Cu(II) center, but very weak for **CuL^2^**. The *T*-dependent susceptibility measurements also show very similar results (*µ*_eff_ = 1.98 µ_B_ for **CuL^1^** and 2.00 µ_B_ for **CuL^2^** and very small Curie–Weiss constants of about −1. The EPR spectra of both complexes show axial symmetry, very similar averaged *g* values of 2.123 and 2.125, respectively, and no hyper-fine splitting.

## 1. Introduction

For the development of new interesting materials based on coordination compounds, polytopic or polyfunctional ligands are interesting candidates [1,2,3,4,5,6,7,8,9,10]. Both types can help to selectively assemble discrete mononuclear or polynuclear complexes [1,2,3,4,7,8,9], clusters [6,7], or coordination polymers [1,2,3,4,5,6,7,8] including metal-organic frameworks (MOFs) [8,10] either by providing more than one coordination site (polytopic) or other functionalities (polyfunctional) enabling secondary interactions such as hydrogen bonds, halogen bonds, π-stacking, or hydrophobic van der Waals interactions [11,12,13,14,15,16]. Additionally, the nature of the ligand combined with the *h*ard and *s*oft *a*cids-and *b*ases principle (HSAB) help in taming the quite flexible geometries of many 3D transition metals. Thus, using the very simple synthesis approach of mixing ligands and transition metal precursors in solution and precipitating or crystallizing the products, defined materials with interesting catalytic [17,18,19,20,21], electrochemical [21,22], magnetic [23,24,25], photochemical [26,27,28,29,30,31,32,33], and photophysical properties [30,31,32,33,34,35] have been obtained in good yields.

Cu(II) is an interesting metal for magnetic materials due to its unpaired spin (*S* = ½) in its d^9^ configuration. The careful design of polyfunctional ligands has allowed to generate dinuclear or oligonuclear Cu(II) coordination compounds with paramagnetic (isolated spins) [24,33], antiferromagnetic (two coupled spins) [23,36], ferromagnetic [37,38], or ferrimagnetic (coupled spins of different species) properties [23,36,39,40,41,42].

Within the vast group of polyfunctional ligands, halogenated pyridines are interesting candidates for the formation of supramolecular materials due to their ability to coordinate to metals using the pyridine function and at the same time potentially form intermolecular halogen bonds to aggregate into supramolecular structures in the solid [14,17,40]. However, only a few Cu(II) complexes or coordination polymers with simple halido-pyridines have previously been reported [43,44,45].

**Scheme 1:**
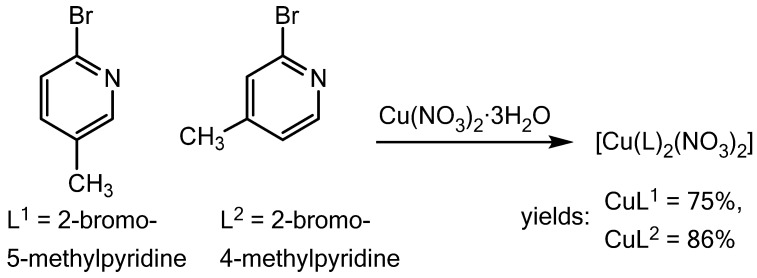
Synthesis and yields of the two Cu(II) complexes of this study.

Herein, we report the reaction of Cu(NO_3_)_2_·3H_2_O with 2-bromo-5-methylpyridine and 2-bromo-4-methylpyridine (Scheme 1) forming the two isomeric complexes [Cu(L)_2_(NO_3_)_2_]. The two compounds show very different structural features (in single crystal X-ray studies and Hirshfeld analyses) which also translate into the optical (UV–vis absorption) and magnetic properties (magnetism and EPR). We also report their FT-IR and Raman characterization and thermal properties.

## 2. Results and Discussion

### 2.1. Syntheses and Analyses

Reactions of Cu(NO_3_)_2_·3H_2_O with 2-bromo-5-methylpyridine (L^1^) in MeCN solution gave the complex [Cu(L^1^)_2_(NO_3_)_2_] (**CuL^1^**) in 75% yield, while the reaction with 2-bromo-4-methylpyridine (L^2^) produced [Cu(L^2^)_2_(NO_3_)_2_] (**CuL^2^**) in 86% yield (Scheme 1, details in the Section 3), both as deep blue crystals. The two stereoisomers showed identical CHN analyses.

### 2.2. Crystal Structures

**Figure 1:**
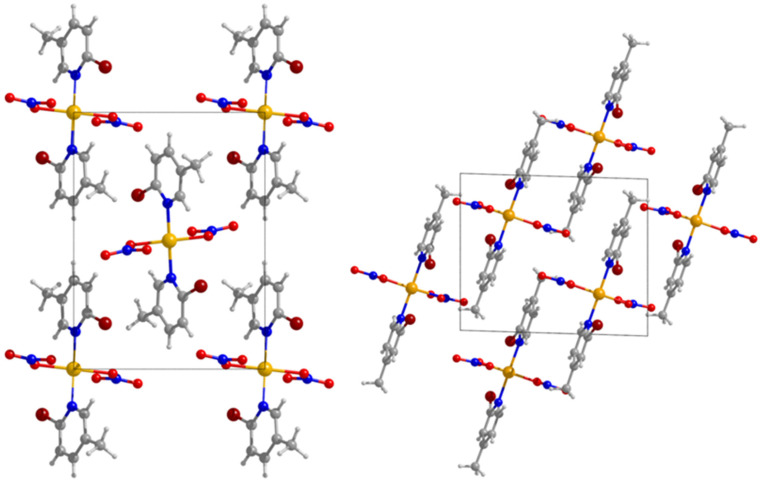
Projection of the crystal structures of [Cu(L)_2_(NO_3_)_2_]. (**left**): **CuL^1^**, L^1^ = 2-bromo-5-methylpyridine complex in *P*2_1_/c; (**right**): **CuL^2^**, L^2^ = 2-bromo-4-methylpyridine derivative in *P*$\bar{1}$, both viewed along the crystallographic *a* axis. Yellow balls represent Cu atoms, light red = O, blue = N, grey = C, white = H, and dark red = Br atoms.

The two isomers of the title compound [Cu(L)_2_(NO_3_)_2_] (L^1^ = 2-bromo-5-methylpyridine or L^2^ = 2-bromo-4-methylpyridine crystallized in two different structures (Figure 1). The 5-methyl isomer **CuL^1^** was solved in the monoclinic space group *P*2_1_/c (Z = 2) with the cell parameters *a* = 6.7738(7) Å, *b* = 13.1890(9) Å, *c* = 10.0999(10) Å, *β* = 103.298(8)°, and a unit cell volume of 878.13(14) Å^3^ (Table S1, Supplementary Materials). The 4-methyl derivative **CuL^2^** was solved and refined in the triclinic space group *P*$\bar{1}$ (Z = 2) with the cell parameters *a* = 7.4916(5) Å, *b* = 10.0404(7) Å, *c* = 12.0723(8) Å, *α* = 86.176(6)°, *β* = 77.769(5)°, *γ* = 79.793(5)°, and a unit cell volume of 873.00(10) Å^3^ (Table S1).

**Figure 2:**
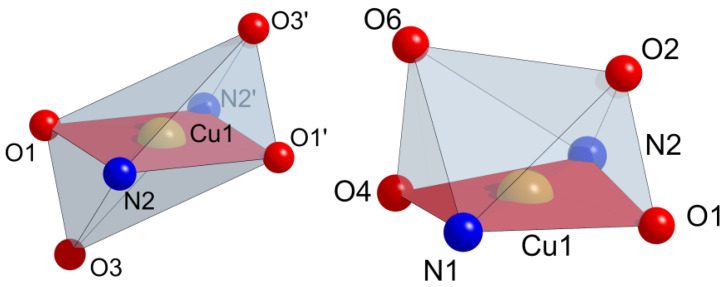
Environment of the Cu atom in the two isomers of [Cu(L)_2_(NO_3_)_2_] with atom numbering. (**left**): **CuL^1^**, L^1^ = 2-bromo-5-methylpyridine in *P*2_1_/c; (**right**): **CuL^2^**, L^2^ = 2-bromo-4-methylpyridine in *P*$\bar{1}$.

**Figure 3:**
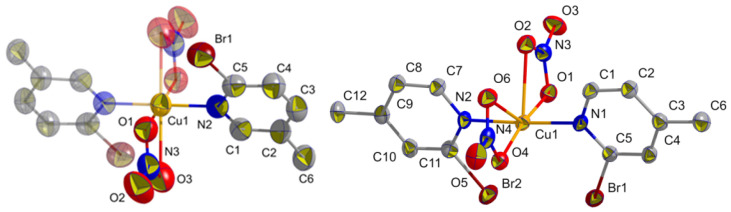
The asymmetric unit of the two isomers of [Cu(L)_2_(NO_3_)_2_] with atom numbering. (**left**): **CuL^1^**, L^1^ = 2-bromo-5-methylpyridine in *P*2_1_/c; (**right**): **CuL^2^**, L^2^ = 2-bromo-4-methylpyridine in *P*$\bar{1}$.

The molecular entities of both forms show the same coordination pattern around the Cu(II) centers, which is *trans* square planar with two pyridine N atoms and two nitrate O atoms contributing to a square planar N_2_O_2_ coordination (Figure 2 and Figure 3; essential metrical data in Table S2). For the 2-bromo-5-methylpyridine complex **CuL^1^** (*P*2_1_/c), two additional O atoms from the coordinated NO_3_^−^ ligands O1 and O1^i^ (with (i) = −x + 2, −y, −z + 2) contribute to an overall elongated octahedral (4 + 2) coordination with Cu⋯O_axial_ distances of 2.515(7) Å (Table S2). The Cu–O bonds are much longer than those of the two equatorial O atoms and due to the small bite angle of the NO_3_^−^ ligand, these two O atoms are not in an ideal position for a perfect tetragonally elongated octahedron (D_4h_). The additional deviation of the two axial ligands distorts the coordination sphere towards an overall C_2v_ symmetry. The tetragonal elongation is frequently found for Cu(II) complexes [46,47,48], this C_2v_ deviation has been previously observed for derivatives with equatorial-axial chelate ligands [46,47]. The previously reported Cu(II) bis-nitrato bis-pyridine complexes [Cu(L)(NO_3_)_2_] (L = 2-chloro-5-methylpyridine [49] and [Cu(4-MTPP)(NO_3_)_2_] (4-MTPP = 4-(methylthio)-4-(pyridin-2-yl)pyrimidine [50] and the bis-pyridine-N-oxide complex [Cu(2,6-dmnpn)_2_(NO_3_)_2_] (2,6-dmnpn = 4-NO_2_-2,6-Me_2_-pyridine-N-oxide) [51] show the same type of bonding with very similar geometry around Cu(II), while [Cu(Py)_2_(NO_3_)_2_]_2_ Py has a dimeric structure with the Cu-bound O_NO3_ atom bridging between the Cu(II) centers [52].

This stands in contrast to the 2-bromo-4-methylpyridine derivative (**CuL^2^**), where two further O atoms of the NO_3_^−^ ligands (O2 and O6) are both localized on the same side of the equatorial N_2_O_2_ plane. Thus, they form a rather unusual double-tipped square pyramidal coordination around Cu(II). However, whilst this arrangement also agrees with the Jahn–Teller distortion of the Cu(II) d^9^ system [47], it is rather exotic. In the case of the 2-bromo-4-methyl ligand, this peculiar orientation of the two NO_3_^−^ ligands is in line with the crystal symmetry (Cu does not lie at the center of the inversion) and goes along with the two Br substituents pointing in the same direction which could be called a *syn* conformation, contrasting with the *anti* conformation of the two Br atoms in the 2-bromo-5-methyl derivative. The Br⋯Br distance of 3.694(3) Å in **CuL^2^** is slightly shorter than the sum of the van der Waals radii of 3.72 Å [53], while the Cu⋯Br distance of about 3.26 Å is far too long to account for coordination (3.33 Å for **CuL^1^**).

In the crystal structure, both forms show a number of intermolecular hydrogen bonds (Table S3). The shortest ones for **CuL^1^** with ~2.58 Å are found between O2 and the H–C4 and H–C6 groups of the pyridine ligand, while for **CuL^2^** slightly shorter intermolecular distances of about 2.39 (O4⋯H–C12), 2.48 (O6⋯H–C12), and 2.49 Å (O5⋯H–C10) were found. All these hydrogen bonds are considered to be weak and essentially of an electrostatic nature [54]. Intermolecular Br⋯Br halogen bonds were not observed, the two Br substituents in **CuL^2^** point towards H–C3 and H–C6 functions of neighboring molecules with Br⋯H contacts longer than 3 Å (Figure S1).

Powder X-ray diffraction (PXRD) showed an excellent match of the experiment with the patterns calculated from the single crystal XRD for **CuL^1^** (Figure S2). The powder diffractogram of **CuL^2^** also matched with the pattern calculated from the single crystal structure but the materials show a lower degree of crystallinity (broader signals) (Figure S3). Nevertheless, the PXRD patterns of both compounds confirm that they are phase-pure and thus suitable for the measurement of thermal, spectroscopic, and magnetic properties.

### 2.3. Hirshfeld Surface Analysis and Energy Framework Calculations

Hirshfeld surface analysis was carried out for a more detailed view on the intermolecular interactions. The important intermolecular interactions in the molecule are highlighted by the *d*_i_ and *d*_e_ plotted as fingerprints. The fingerprint plot analysis for **CuL^1^** showed that intermolecular H⋯O interactions make the highest contribution with 44.6% of the total, while others are markedly smaller 14.6% (H⋯Br), 12.5% (H⋯H), 12.4% (C⋯H), and 5% (N⋯H), respectively (Figure S4). For **CuL^2^**, the O⋯H interactions account for 41.2% which is slightly lower than the 44.6% for the **CuL^1^** derivative and stands somewhat in contrast to the shorter hydrogen bonds found in the crystal structures for the **CuL^2^** complex. However, this analysis summarizes the interactions and does not weight them. The Br⋯H (15.9%), H⋯H (13.4%), C⋯H (9.4%), and N⋯H (4.8%) interactions are also very similar.

An energy framework analysis [55] was carried out for the two compounds (Figure S5, Table S4). The interaction energies include electrostatic (E′_ele(L_^1^_)_ = −39.9 kJ mol^−1^; E′_ele(L_^2^_)_ = −168.6 kJ mol^−1^), polarization (E′_pol(L_^1^_)_ = −33 kJ mol^−1^; E′_pol(L_^2^_)_ = −144.1 kJ mol^−1^), dispersion (E′_dis (L_^1^_)_ = −120.6 kJ mol^−1^; E′_dis(L_^2^_)_ = −269 kJ mol^−1^), and repulsion (E′_rep(L_^1^_)_ = 58.9 kJ mol^−1^; E′_rep(L_^2^_)_ = 157.7 kJ mol^−1^) contributions with a total interaction energy of E_tot(L_^1^_)_ = −122.7 kJ mol^−1^ for **CuL^1^** and E_tot(L_^2^_)_ = −340.4 kJ mol^−1^ for **CuL^2^**. When comparing the individual contributions, it becomes clear that all of them are higher for **CuL^2^** (or lower for the repulsion) due to the lower symmetry. However, concerning the resulting marked difference in total energy of −122.7 kJ mol^−1^ for **CuL^1^** vs. −340.4 kJ mol^−1^ for **CuL^2^**, we are skeptical, because the thermal properties and the IR and Raman spectroscopy of the two complexes are not very different (see later). In future work, we will calculate the stability of the two complexes in the crystal lattice in more detail using density functional theory (DFT) methods.

### 2.4. Infrared and Raman Spectroscopy

**Figure 4:**
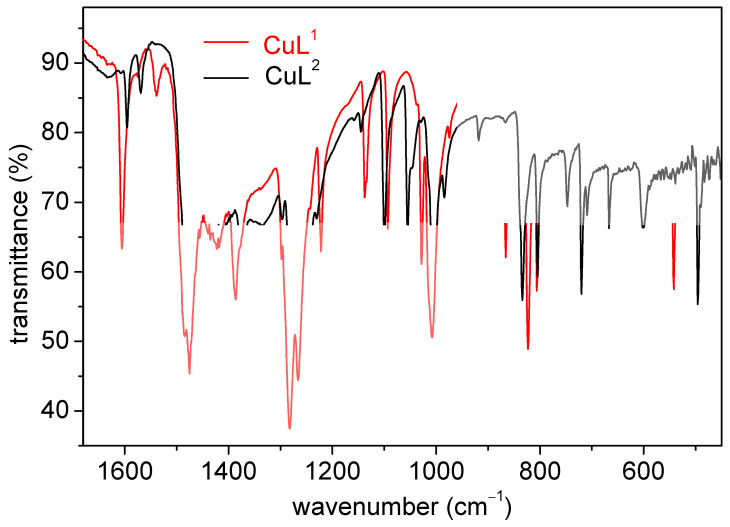
FT-IR spectra of the complexes **CuL^1^** (L^1^ = 2-bromo-5-methylpyridine) (red) and **CuL^2^** (L^2^ = 2-bromo-4-methylpyridine (black).

The asymmetric and symmetric H–C vibrations of the CH_3_ and CH groups of both complexes were observed in the FT-IR spectra in the range from 3150 to 2850 cm^−1^ followed by aromatic H–C overtones in the 2800–1700 cm^−1^ range (Figure S6) [56,57,58,59,60]. When comparing the two complexes **CuL^1^** and **CuL^2^** (L^1^ = 2-bromo-5-methylpyridine, L^2^ = 2-bromo-4-methylpyridine), similar resonances were found in the range from 1600 to 120 cm^−1^ (Figure 4). The underlying pyridine C=C and C=N and NO_3_ stretching frequencies were generally at a higher energy for the **CuL^1^** complex and the relative intensities differ between the two stereoisomers with their different coordination pattern. The same is true for the umbrella-like H–C vibration modes of the CH_3_ substituents, the H–C arene oscillations, the H–C wagging, and the C–Br stretches [56,57,58] in the range from 1150 to 900 cm^−1^. Even more different are the Raman spectra of the two complexes (Figure S7). However, here all the characteristic bands are shifted to a lower energy for the **CuL^1^** complex compared with the **CuL^2^** derivative especially the C–Br stretches at around 800 cm^−1^ and the NO_3_ stretches at around 700 cm^−1^ which are also found in the FT-IR. Additionally, the Cu–N and Cu–O stretches in the range between 400 and 200 cm^−1^ are shifted to lower energy for the **CuL^1^** derivative which would mean slightly weaker Cu–ligand bonds. However, the thermal analyses (TG/DTA) do not show marked differences in their behavior (see below) and the different *T* of the single crystal X-ray diffraction data precludes comparison.

### 2.5. Thermal Analysis

Thermogravimetric (TG) analyses shows that the two compounds are thermally stable up to about 160 °C (Figure S8). The differential thermal analysis (DTA) shows an endothermic peak at around 200 °C for both complexes (slightly higher for **CuL^2^**), followed by one or two intense exothermic signals in the range 220 to 300 °C. We assume the decomposition of the nitrate ligands to NO, NO_2_, and O_2_ being responsible for these exothermic peaks. The final mass loss after heating to 300 °C is 83% for **CuL^1^** and 86% for **CuL^2^**, which is in line with the formation of CuO (calculated loss of 85%). The same behavior was observed for comparable Cu(II) complexes [60,61,62,63].

### 2.6. UV–Vis Absorption Spectroscopy

**Figure 5:**
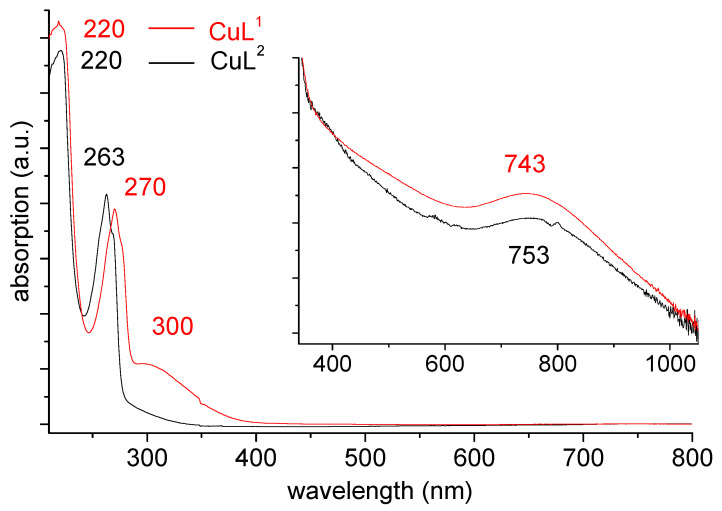
UV–vis absorption spectra of the complexes **CuL^1^** (L^1^ = 2-bromo-5-methylpyridine and **CuL^2^**, L^2^ = 2-bromo-4-methylpyridine, in EtOH solution. Conc. for the main spectra = 10^−4^ mol/L; conc. for the insert = 10^−3^ mol/L).

The two complexes show very similar absorption spectra in EtOH solution. An intense band around the 220 nm is identical for both complexes (Figure 5). Marked differences were found in the UV–vis range where the **CuL^1^** complex shows a maximum at 270 nm, while the same band for the **CuL^2^** derivative is found at 263 nm. These bands can be assigned to π–π* transition as they also occur for the ligands (Figure S9). For the **CuL^1^** complex, we observed a broad shoulder in the range from 300 to 350 nm which we assign to a ligand_(NO3)_-to-metal-charge transfer excitation. For the **CuL^2^** derivative, this is far less pronounced which we ascribe to the unusual orientation of the two NO_3_^−^ ligands compared to **CuL^1^**. The long-wavelength absorptions at around 750 nm are quite similar for both complexes (743 nm for **CuL^1^**, 753 nm for **CuL^2^**). They were assigned to the ^2^*B*_2g_ → ^2^*E*_g_ and ^2^*A*_1g_ → ^2^*E*_g_ electronic transitions in Cu(II), d^9^ systems [29,32,46,59,63]. They are responsible for the typical blue color of the two Cu(II) complexes.

While the similar NIR bands might point to a decomposition of the complexes in EtOH solution, forming the non-coordinated ligands and a [Cu(II)(EtOH)_n_]^2+^ solvate, the bands in the 300–350 nm range which do not occur for the ligands, support that the complexes were stable in EtOH solution. If that is the case, we conclude that the NIR absorptions of the complexes are either not affected by the different coordination of the two isomers, or **CuL^2^** forms the same *anti* structure as **CuL^1^** in solution. In future work, we will study this possibility in more detail.

### 2.7. Magnetization and EPR Studies

**Figure 6:**
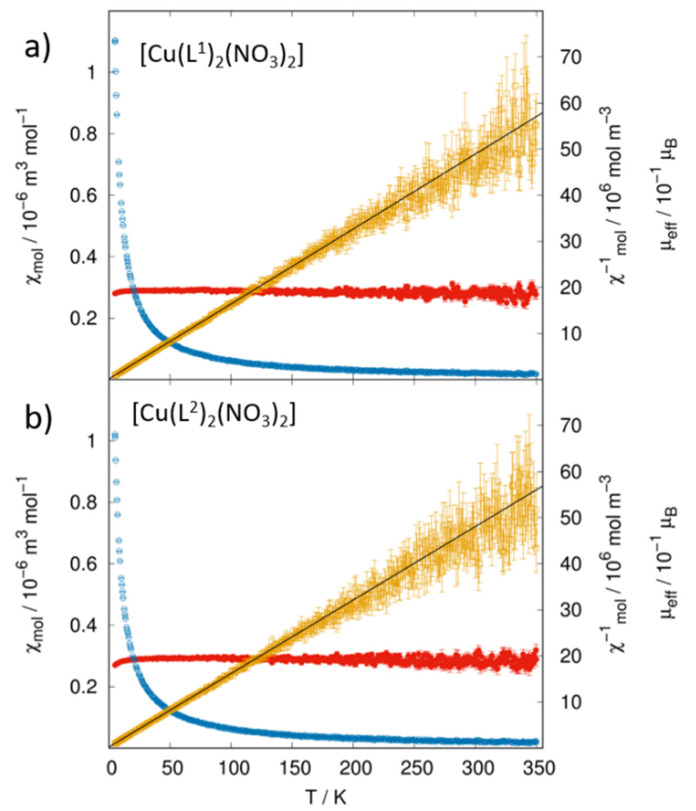
Molar susceptibilities (blue circles) and inverse molar susceptibilities (orange squares) of (**a**) **CuL^1^** and (**b**) **CuL^2^** with fits according to the Curie–Weiss law (black solid line). The red curve represents the effective magnetic moments (in µ_B_).

Temperature-dependent magnetic susceptibility data (Figure 6) reveal a nearly perfect paramagnetic behavior in the entire temperature range from 5 to 350 K, in line with the isolated Cu(II) centers. A Curie–Weiss fit of the inverse magnetic susceptibility yields nearly identical effective magnetic moments of *µ*_eff_ = 1.98 µ_B_ for **CuL^1^** and *µ*_eff_ = 2.00 µ_B_ for **CuL^2^**. Both are constant over a wide temperature range and within the range from 1.7 to 2.2 µ_B_ experimentally observed for similar Cu(II) complexes at room temperature [25,64,65,66,67,68,69,70]. The effective moments being larger than the spin-only value of 1.73 µ_B_ expected for a 3d^9^ configuration (*S* = 1/2) indicate spin-orbit coupling [23,37,39,64,65].

Based on *S* = 1/2 we calculated *g* values of 2.29 for **CuL^1^** and 2.32 for **CuL^2^** from the magnetic moments. The azido-bridged coordination polymers [Cu(4-HOCH_2_py)(μ_1,1_-N_3_)(μ_1,3_-N_3_)]_n_ and [Cu(2-Br-4-Mepy)(μ^1,1^-N_3_)_2_]_n_ with five-coordinated Cu(II) showed a similar *T*-independent behavior as **CuL^1^** and **CuL^2^** and their *g* values of 2.22 and 2.09, respectively, are also in the same range. The authors assign this to antiferromagnetic coupling through the bridging azide ligands [68]. For the mononuclear complex [Cu(L)_2_Cl_2_] (L = pyridine-4-carboxamido-2-methyl-phenyl-4-methyl ester) with a square planar Cu(II) a smaller *g* value of 2.13 was reported [69], while for the mononuclear pyridine-*N*-oxide complex [Cu(C_5_H_5_NO)_2_(NO_3_)_2_] with a similar coordination as **CuL^1^** and **CuL^2^** a *g* value of 2.17 was reported [66].

The Weiss constants of −1 K (**CuL^1^**) and −1.5 K (**CuL^2^**) are comparably small. A Weiss constant >0 indicates dominating ferromagnetic interactions and for <0 dominating antiferromagnetic interactions [23,64]. Thus, we can attribute a weak antiferromagnetic character to both complexes. For the pyridine-*N*-oxide Cu(II) complex [Cu(C_5_H_5_NO)_2_(NO_3_)_2_] a very similar Weiss constant of −0.8 K was reported [66] and the values for the azido-bridged coordination polymers [Cu(4-HOCH_2_py)(μ_1,1_-N_3_)(μ^1,3^-N_3_)]_n_ (−1.3 K) and [Cu(2-Br-4-Mepy)(μ^1,1^-N_3_)_2_] (−1.1 K) are only slightly smaller [68] in line with their small antiferromagnetic coupling. The similar *T*-independent behavior (see above) and the very similar Weiss constants for **CuL^1^**, **CuL^2^**, and these azido-bridged complexes is interesting as in the azido-bridged complexes the antiferromagnetic coupling is promoted through the bridging azido ligands, while in **CuL^1^** and **CuL^2^**, no such direct interactions between the Cu(II) centers occur. In contrast to this, a Weiss constant of −9.31 K was reported for the tetrachlorido cuprate(II) [Cu(bix)Cl_4_]·H_2_O (bix = 1,4-bis(imidazol-1-ylmethyl)benzene) [67] which is likely due to strong O–H⋯Cl hydrogen bonds connecting the tetrahedral [CuCl_4_]^2−^ units in the structure. Thus, both *g* values and the Weiss constants from magnetic measurements do not allow unequivocal conclusions on the structures of Cu(II) complexes.

**Figure 7:**
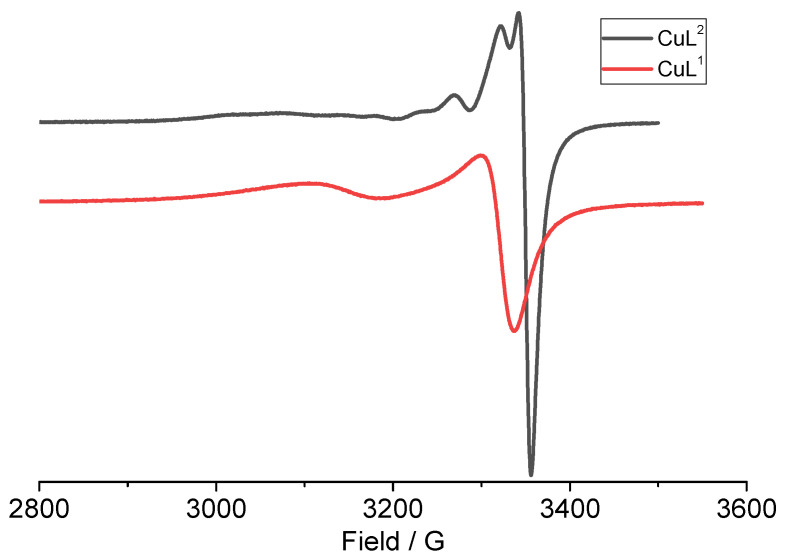
X-band EPR spectra of the two Cu(II) complexes [Cu(L)_2_(NO_3_)_2_], recorded on solid samples at frequencies of 9.642502 GHz for **CuL^2^** (L^2^ = 2-bromo-4-methylpyridine) and 9.645555 GHz for **CuL^1^** (L^1^ = 2-bromo-5-methylpyridine) at 298 K. Receiver gain of 40, attenuation 25 dB (0.3162 mW), 1 scan for both samples.

The X-band EPR spectra of a powder sample of **CuL^1^** showed an axial spectrum (Figure 7) with no hyperfine splitting (HFS). The spectrum was simulated (Figure S10) using the *g* values *g*_‖_ = 2.230 and *g*_⊥_ = 2.070, which average to *g*_av_ = 2.123, the *g* anisotropy ∆*g* calculates to 0.160 (Table 1).

In contrast to this, we observed two signals for **CuL^2^** (L^2^ = 2-bromo-4-methylpyridine) (Figure 7; Figure S11). Simulations allowed to identify one species with similar parameters as the **CuL^1^** complex, *g*_‖_ = 2.260 and *g*_⊥_ = 2.057, which averages to *g*_av_ = 2.125. The *g* anisotropy ∆*g* for this species calculates to 0.203 (species I in Table 1). We conclude that this spectrum represents **CuL^2^**, which is supported by the missing HFS in both spectra. The spectrum of the second species was simulated using *g*_‖_ = 2.135 and *g*_⊥_ = 2.057, which averages to *g*_av_ = 2.083 The *g* anisotropy ∆*g* calculates to 0.078 (species II in Table 1). Here, pronounced HFS of ^63/65^Cu (*I* = 3/2) and ^14^N (*I* = 1) was observed. At the moment, we have no idea about the nature of the second Cu(II) species and how the PXRD failed to detect it in the **CuL^2^** product. It may be that the EPR sample of **CuL^2^** contained molecules of both *syn* and *anti* conformations in the same crystal structure. Such *syn*-to-*anti* conversion was already discussed for the UV–vis absorption spectroscopy in solution. To observe this in the solid would be even more fascinating and we will study this in detail in the near future.

The *g* values derived from the magnetic measurements of 2.29 for **CuL^1^** and 2.32 for **CuL^2^** both lie higher than the averaged *g* values from EPR spectroscopy but both values are higher for **CuL^2^** while the values from the magnetic measurements roughly coincide with the *g*_‖_ values from EPR. We attribute these differences to the specific geometry and orientation of crystallites in the EPR experiment.

For the geometrically very similar complex [Cu(2,6-dmnpn)_2_(NO_3_)_2_] (2,6-dmnpn = 4-NO_2_-2,6-Me_2_-pyridine-N-oxide) [51], three *g* values *g*_z_ = 2.295, *g*_y_ = 2.086, *g*_x_ = 2.066 were reported. Overall, the spectrum looked very similar to those of **CuL^1^** and **CuL^2^** with a slightly different *g*_av_ = 2.149 which is probably due to the O–Cu bonding of the pyridine-N-oxide ligands, but a Δ*g* = 0.229 which is very similar to our values. We have recently shown that EPR spectra of such pseudo-axial geometry (*g*_y_ and *g*_x_ very similar) are frequently observed for Cu(II) complexes containing pyridine-amide ligands. Very similar *g*_av_ values of over 2.1, Δ*g* values of around 0.2 and no HFS are in line with various tetragonally elongated octahedral structures [46]. Although the local symmetry around Cu(II) is very different for **CuL^1^** and **CuL^2^**, EPR spectroscopy does not allow for tracing of the two very different distortion variants from the ideal octahedral geometry.

## 3. Experimental Section

### 3.1. Chemicals

Cu(NO_3_)_2_·3H_2_O was purchased from Fluka (Fisher Scientific, Schwerte, Germany), 2-bromo-5-methylpyridine and 2-bromo-4-methylpyridine were purchased from BLDpharm (BLDpharm, Kaiserslautern, Germany) and used without further purification.

### 3.2. Syntheses

#### 3.2.1. Synthesis of [Cu(L^1^)_2_(NO_3_)_2_] L^1^ = 2-Bromo-5-methylpyridine

A total of 0.241 g (1 mmol) of Cu(NO_3_)_2_·3H_2_O was dissolved in 3 mL of MeCN and added to 3 mL of MeCN solution containing 0.170 g (1 mmol) 2-bromo-5-methylpyridine. The mixture was stirred for 3 h and then a blue solution was filtered from a blue solid residue. From the blue solution, crystals were obtained through slow evaporation at ambient conditions. Yield: 403 mg (0.75 mmol, 75%) of blue crystals. Elemental analysis (C_12_H_12_Br_2_CuN_4_O_6_): calcd. (found), C 27.11 (27.10), H 2.28 (2.25), N 10.54 (10.55)%. FT-IR: 3095, 3026, 1596, 1380, 1478, 1277, 995, 830, 750, 494 cm^−1^. UV–vis (in EtOH): 220, 270, 300sh, 743 nm.

#### 3.2.2. Synthesis of [Cu(L^2^)_2_(NO_3_)_2_] L^2^ = 2-Bromo-4-methylpyridine

A total of 0.126 g (0.52 mmol) of Cu(NO_3_)_2_·3H_2_O was dissolved in 2 mL of EtOH and added to 2 mL of EtOH solution containing 90 mg (0.52 mmol) of 2-bromo-4-methylpyridine. The mixture was stirred for 3 h and left for evaporation at ambient temperature. Yield: 460 mg (0.86 mmol, 86%) of blue crystals. Elemental analysis (C_12_H_12_Br_2_CuN_4_O_6_): calcd. (found), C 27.11 (27.09), H 2.28 (2.30), N 10.54 (10.51)%. FT-IR: 3110, 3076, 1606, 1455, 1387, 1280, 1003, 830, 703, 534 cm^−1^. UV–vis (in EtOH): 220, 263 nm, 753 nm.

### 3.3. Instrumentation

Elemental analyses were carried out using a Hekatech CHNS EuroEA 3000 Analyzer (Hekatech, Wegberg, Germany). Raman measurement was carried out using the Renishaw inVia Reflex Raman (Renishaw, Kingswood, UK). TG measurements were performed using a simultaneous Mettler Toledo TGA/DSC 2/1600 thermal analyzer (Mettler Toledo, Barcelona, Spain) in an N_2_ atmosphere at a heating rate of 10 K min^−1^. Fourier-transformed infrared (FT-IR) spectra on KBr pellets were obtained using a PerkinElmer UATR II spectrometer (Perkin Elmer, Waltham, MA, USA) in the range from 4000 to 400 cm^−1^ at room temperature. UV–vis absorption spectra were recorded in EtOH using a SCINCO S-3100 spectrophotometer (Scinco, Seoul, Republic of Korea) or a Varian Cary 05E spectrophotometer (Varian, Darmstadt, Germany). EPR spectroscopy was measured on solid samples in the X-band using a Bruker EMXNano spectrometer (Bruker, Rheinhausen, Germany) at 298 K; *g* values were calibrated using dpph.

### 3.4. Magnetization Measurements

The magnetic properties were studied using a Quantum Design PPMS Evercool II (Quantum Design GmbH, Darmstadt, Germany) with vibrating sample magnetometer (VSM) option. Then, 12.8 mg of **CuL^1^** and 10.1 mg of **CuL^2^** of powdered samples were enclosed in commercially available polypropylene powder capsules and fixed in a brass sample holder. The temperature-dependent magnetization was measured after cooling in an applied field of 10 mT in a temperature range from 5 to 350 K with a heating rate of 1 K min^−1^. A small background signal resulting from the sample holder was determined from reference measurements and subtracted from the measured sample data. The obtained susceptibility data were corrected for a diamagnetic susceptibility of *χ*_dia_ = −271.88 × 10^−11^ m^3^ mol^−1^ [64]. A fit of the inverse molar susceptibility data according to the Curie–Weiss law was carried out in the temperature range from 20 to 220 K.

### 3.5. Single-Crystal X-ray Diffraction

The x-ray singe crystals structure analyses were carried out at 293(2) K for [Cu(L^1^)_2_(NO_3_)_2_] and at 150(2) K for [Cu(L^2^)_2_(NO_3_)_2_] with a Stoe IPDS 2T diffractometer and equipped with graphite-monochromatized Mo-Kα radiation. The structures were solved by using SHELXT2018/3 [71] and refined using SHELXL [72,73]. A summary of the crystal data, experimental details, and refinement results are provided in Table S1. CCDC 2207776 for [Cu(L^1^)_2_(NO_3_)_2_] (L^1^ = 2-bromo-5-methylpyridine) and 2207769 for [Cu(L^2^)_2_(NO_3_)_2_] (L^2^ = 2-bromo-4-methylpyridine) contain the supplementary crystallographic data for this paper. These data can be obtained free of charge at www.ccdc.cam.ac.uk/conts/retrieving.html (accessed on 4 December 2022) or from the Cambridge Crystallographic Data Centre, Cambridge, 12 Union Road, Cambridge, CB2 1EZ UK. Fax: +44-1223-336-033; Email: deposit@ccdc.cam.ac.uk.

### 3.6. Powder X-ray Diffraction

PXRD data were collected at room temperature on a Rigaku *MiniFlex 600-C* powder diffractometer, Cu K*α* radiation (λ = 1.54186 Å, Ni filter, 293(2) K, 3° ≤ 2θ ≤ 90°), D/teX Ultra silicon strip detector. Typical recording times were approx. 20 min with a step size of 0.005° (2θ). Employing the WinXPow software suite (WinXpow, version 3.12, Stoe & Cie GmbH, Darmstadt, Germany the recorded patterns were compared with theoretical patterns simulated from known single-crystal structure data.

### 3.7. Hirshfeld Surface Analysis

Hirshfeld surface analysis, their relative 2D fingerprint plots, and the energy framework calculations are an essential technique for showing packing modes and intermolecular interactions of crystals’ structure using a CIF file in the Crystal Explorer 17 software [74]. The quantifying and decoding of the inter molecular contacts in the crystal packing are visualized using *d*_norm_ (normalized contact distance) and 2D fingerprint plots, respectively. The dark-red spots on the *d*_norm_ surface arise as a result of short interatomic contacts, while the other inter molecular interactions appear as light-red spots. *d*_i_ (inside) and *d*_e_ (outside) represent the distances to the Hirshfeld surface from the nuclei, with respect to the relative van der Waals radii. The proportional contribution of the contacts over the surface is visualized by the color gradient (blue to red) in the fingerprint plots. The energy framework calculations are estimated from a single point molecular wave function at HF/3-21G. A cluster of 3.8 Å radius was generated around the molecule and the energy calculation was performed. The neighboring molecules (density matrices) are generated within this shell by applying crystallographic symmetry operations with respect to the central molecule (density matrix). The interaction energy is broken down as *E*_tot_ = *k*_ele_*E′*_ele_ + *k*_pol_E′_pol_ + *k*_dis_*E*′_dis_ + *k*_rep_*E*′_rep_ where the k values are scale factors, *E′*_ele_ represents the electrostatic component, *E′*_pol_ the polarization energy, *E*′_dis_ the dispersion energy, and *E*′_rep_ the exchange–repulsion energy [55,75,76].

## 4. Conclusions

Reacting Cu(NO_3_)_2_·3H_2_O with the two isomeric 2-bromo-5-methylpyridine (L^1^) and 2-bromo-4-methylpyridine (L^2^) ligands led to two isomeric 2-bromomethylpyridine Cu(II) complexes [Cu(C_6_H_9_NBr)_2_(NO_3_)_2_] as air-stable blue materials in good yields. The x-ray single crystal diffraction showed different crystal structures ([Cu(L^1^)_2_(NO_3_)_2_] (**CuL^1^**) in *P*2_1_/c, [Cu(L^2^)_2_(NO_3_)_2_] (**CuL^2^**) in *P*$\bar{1}$). The molecular structure showed a primary *trans*-*N*_2_(Py)*O*_2_(NO_3_) coordination of the Cu(II) centers for both complexes but a very different orientation of the Br substituents. The expected structure, which we described as *anti* was found for **CuL^1^** while **CuL^2^**, showed an unusual *syn* conformation with neighboring Br atoms and rather short Br⋯Br contacts of about 3.7 Å. Moreover, **CuL^1^** showed the expected 4+2 distorted coordination with the two secondary (axially elongated) Cu–O bonds from the NO_3_ ligands on each side of the CuN_2_O_2_ basal plane, while for **CuL^2^** both secondary O atoms lie on the side which is not occupied by the Br substituents. While the general Hirshfeld surface analysis gave very similar results for both structures, an energy framework analysis gave superior stability for **CuL^2^** due to the lower symmetry. In contrast to this, the thermal stability (TG/DTA) of both complexes is almost the same with decomposition starting at around 160 °C. The FT-IR and Raman frequencies are markedly different for the two isomers. The *T*-dependent susceptibilities again show very similar results (*µ*_eff_ = 1.98 µ_B_ for **CuL^1^** and 2.00 µ_B_ for **CuL^2^** and very small Curie–Weiss constants of about −1 K. The EPR spectra of both complexes show axial symmetry, very similar averaged *g* values of 2.123 and 2.125, respectively, and no hyper-fine splitting. The only difference here lies in the markedly higher *g* anisotropy Δ*g* of 0.203 for **CuL^2^** compared with 0.160 for **CuL_1_**. In the fluid EtOH solution, the two complexes seem to be stable and UV–vis absorption spectra show marked differences in the π–π* absorptions at 263 for **CuL^2^** and 270 for **CuL^1^** nm and in the ligand-to-metal charge transfer bands at around 320 nm which are pronounced for **CuL^1^** but very weak for **CuL^2^**. We assign this difference to the higher symmetry at the Cu(II) center for **CuL^1^**. In contrast to this, the long-wavelength d–d* bands at around 750 nm are very similar for both complexes. At the same time, we cannot rule out that **CuL^2^** is present in solution in the same isomeric *anti* form as **CuL_1_** and that only lattice energies are responsible for the unexpected structure of **CuL^2^** in the solid. In future work, we will gauge the stability of the two isomeric forms by sophisticated DFT calculations as well as by UV–vis absorption and EPR spectroscopy.

## Figures and Tables

**Table 1:** X-band EPR data of the two complexes [Cu(L)_2_(NO_3_)_2_] ^a^.

	*g*_‖_	*g*_⊥_	*g*_av_	∆*g*	HFS ^b^	Line Width ^c^
[Cu(L^1^)_2_(NO_3_)_2_]	2.230	2.070	2.123	0.160	none	50, 27, 27
[Cu(L^2^)_2_(NO_3_)_2_] species I	2.260	2.057	2.125	0.203	none	95, 22, 22
species II	2.135	2.057	2.083	0.078	*A*_‖N/Cu_ = 40 G	15, 10, 10

^a^ Powdered samples at 298 K, L^1^ = 2-bromo-5-methylpyridine, L^2^ = 2-bromo-4-methylpyridine. Averaged *g* value *g_av_* = (*g_‖_* + 2*g*_⊥_)/3; *g* anisotropy ∆*g* = *g*_‖_ − *g*_⊥_. ^b^ Hyperfine splitting (HFS) in G. ^c^ Simulated line width in G.

## Data Availability

Available from the authors on request.

## Supplementary Materials

The following supporting information can be downloaded at: https://www.mdpi.com/article/10.3390/molecules28020731/s1, Figure S1: Views on the crystal structure of [Cu(L^2^)_2_(NO_3_)_2_] L^2^ = 2-bromo-4-methylpyridine along the crystallographic *c* and *b* axes; Figure S2: X-ray powder diffractogram of [Cu(L^1^)_2_(NO_3_)_2_] L^1^ = 2-bromo-5-methylpyridine measured with Cu-K_α_ radiation in reflection geometry with the calculated pattern for the corresponding single crystal structure; Figure S3: X-ray powder diffractogram of [Cu(L^2^)_2_(NO_3_)_2_] L^2^ = 2-bromo-4-methylpyridine measured with Cu-K_α_ radiation in reflection geometry with the calculated pattern for the corresponding single crystal structure; Figure S4: View of the Hirshfeld surfaces mapped over *d*_norm_ and Hirshfeld two-dimensional fingerprint plots for [Cu(L^1^)_2_(NO_3_)_2_] and [Cu(L^2^)_2_(NO_3_)_2_]; Figure S5: Energy frameworks constructed for Coulomb energy, dispersion energy and total energy for the both compounds [Cu(L^1^)_2_(NO_3_)_2_] L^1^ = 2-bromo-5-methylpyridine in *P2_1_/c* and [Cu(L^2^)_2_(NO_3_)_2_] L^2^ = 2-bromo-4-methylpyridine in *P*$\bar{1}$; Figure S6: FT-IR spectra of the complexes [Cu(L^1^)_2_(NO_3_)_2_] L^1^ = 2-bromo-5-methylpyridine in *P2_1_/c* and [Cu(L^2^)_2_(NO_3_)_2_] L^2^ = 2-bromo-4-methylpyridine in *P*$\bar{1}$; Figure S7: Raman spectra of the complexes [Cu(L^1^)_2_(NO_3_)_2_] L^1^ = 2-bromo-5-methylpyridine in *P2_1_/c* and [Cu(L^2^)_2_(NO_3_)_2_] L^2^ = 2-bromo-4-methylpyridine in *P*$\bar{1}$; Figure S8: TG-DTA curves of [Cu(L^1^)_2_(NO_3_)_2_] and [Cu(L^2^)_2_(NO_3_)_2_]; Figure S9: UV–vis absorption spectra of the ligands L^1^ = 2-bromo-5-methylpyridine and L^2^ = 2-bromo-4-methylpyridine in EtOH; Figure S10: X-band EPR spectrum of [Cu(L^1^)_2_(NO_3_)_2_], (L^1^ = 2-bromo-5-methylpyridine) at 9.645555 GHz and 298 K with simulation; Figure S11: X-band EPR spectrum of [Cu(L^2^)_2_(NO_3_)_2_], (L^2^ = 2-bromo-4-methylpyridine) at 9.642502 GHz and 298 K with simulation; Table S1: Structure solution and refinement data for the two isomeric forms of [Cu(L)_2_(NO_3_)_2_]; Table S2: Selected metrical data of the isomeric forms of [Cu(L)_2_(NO_3_)_2_]; Table S3: Hydrogen bond details of the isomeric forms of [Cu(L)_2_(NO_3_)_2_]; Table S4: Interaction energies (kJ/mol) of the molecular pairs calculated from energy framework calculation of [Cu(L^1^)_2_(NO_3_)_2_] and [Cu(L^2^)_2_(NO_3_)_2_].
