# Supplementary material for: Structure, Optical and Magnetic Properties of Two Isomeric 2-Bromomethylpyridine Cu(II) Complexes [Cu(C6H9NBr)2(NO3)2] with Very Different Binding Motives
Source: Molecules. 2023 Jan 11;28(2):731. doi: 10.3390/molecules28020731 (PMC9866386; doi:10.3390/molecules28020731)
Supplement: Supplementary file 1 [file molecules-28-00731-s001.zip › molecules-2129571-supplementary.pdf]

Supplementary Materials

for

Structures, optical and magnetic properties of two isomeric 2-bromomethyl pyridine Cu(II) complexes [Cu($C_6H_9N_{Br}$)$_2$(NO_3)$_2$] with very different binding motives

Fatma Garci [a,b], Hammouda Chebbi [a,c,*], Nahal Rouzbeh [d], Leonhard Rochels [d], Sabrina Disch [d], Alexander Haseloer [b], Sean S. Sebastian [b], Uwe Ruschewitz [b], Eric Tobechukwu Anthony [b], Axel Klein [b,*], Mohamed Faouzi Zid [a,*]

Contents

Figure S1. Views on the crystal structure of [Cu(L^2)$_2$(NO_3)$_2$] (L^2 = 2-bromo-4-methylpyridine) along the crystallographic *b* axis.
Figure S2. X-ray powder diffractogram of [Cu(L^1)$_2$(NO_3)$_2$] (L^1 = 2-bromo-5-methylpyridine) measured with Cu-K_α radiation in reflection geometry with the calculated pattern for the corresponding single crystal structure.
Figure S3. X-ray powder diffractogram of [Cu(L^2)$_2$(NO_3)$_2$] (L^2 = 2-bromo-4-methylpyridine) measured with Cu-K_α radiation in reflection geometry with the calculated pattern for the corresponding single crystal structure.
Figure S4. View of the Hirshfeld surfaces mapped over d_{norm} and Hirshfeld two-dimensional fingerprint plots for [Cu(L^1)$_2$(NO_3)$_2$] and [Cu(L^2)$_2$(NO_3)$_2$].
Figure S5. Energy frameworks constructed for Coulomb energy, dispersion energy and total energy for the both compounds [Cu(L^1)$_2$(NO_3)$_2$] (L^1 = 2-bromo-5-methylpyridine) in $P2_1/c$ and [Cu(L^2)$_2$(NO_3)$_2$] (L^2 = 2-bromo-4-methylpyridine) in $P\bar{1}$.
Figure S6. FT-IR spectra of the complexes [Cu(L^1)$_2$(NO_3)$_2$] (L^1 = 2-bromo-5-methylpyridine) in $P2_1/c$ and [Cu(L^2)$_2$(NO_3)$_2$] (L^2 = 2-bromo-4-methylpyridine) in $P\bar{1}$.
Figure S7. Raman spectra of the complexes [Cu(L^1)$_2$(NO_3)$_2$] (L^1 = 2-bromo-5-methylpyridine) in $P2_1/c$ and [Cu(L^2)$_2$(NO_3)$_2$] (L^2 = 2-bromo-4-methylpyridine) in $P\bar{1}$.
Figure S8. TG-DTA curves of [Cu(L^1)$_2$(NO_3)$_2$] and [Cu(L^2)$_2$(NO_3)$_2$].
Figure S9. UV-vis absorption spectra of the ligands L^1 = 2-bromo-5-methylpyridine and L^2 = 2-bromo-4-methylpyridine in EtOH.
Figure S10. X-band EPR spectrum of [Cu(L^1)$_2$(NO_3)$_2$], (L^1 = 2-bromo-5-methylpyridine) at 9.645555 GHz and 298 K with simulation.
Figure S11. X-band EPR spectrum of [Cu(L^2)$_2$(NO_3)$_2$], (L^2 = 2-bromo-4-methylpyridine) at 9.642502 GHz and 298 K with simulation.

Table S1. Structure solution and refinement data for the two isomeric forms of [Cu(L)$_2$(NO_3)$_2$].
Table S2. Selected metrical data of the isomeric forms of [Cu(L)$_2$(NO_3)$_2$].
Table S3. Hydrogen bond details of the isomeric forms of [Cu(L)$_2$(NO_3)$_2$].
Table S4. Interaction energies (kJ/mol) of the molecular pairs calculated from energy framework calculation of [Cu(L^1)$_2$(NO_3)$_2$] and [Cu(L^2)$_2$(NO_3)$_2$].

Supplementary Figures

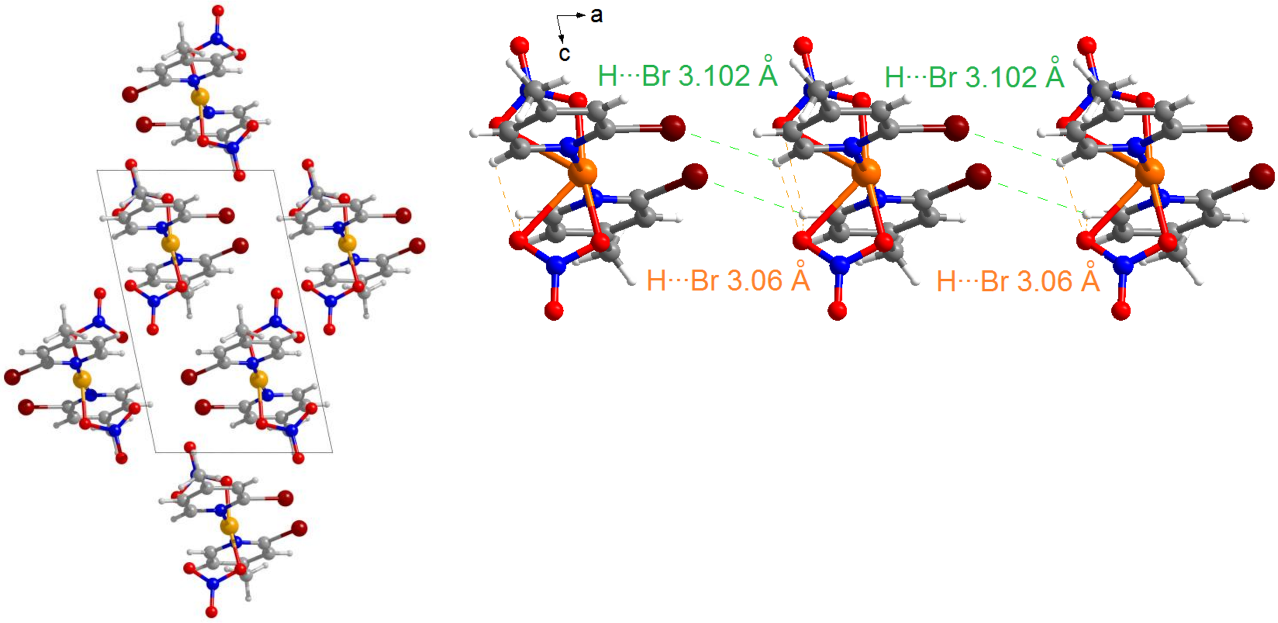

Figure S1. Views on the crystal structure of $[Cu(L^2)_2(NO_3)_2]$ (L^2 = 2-bromo-4-methylpyridine) along the crystallographic *b* axis.

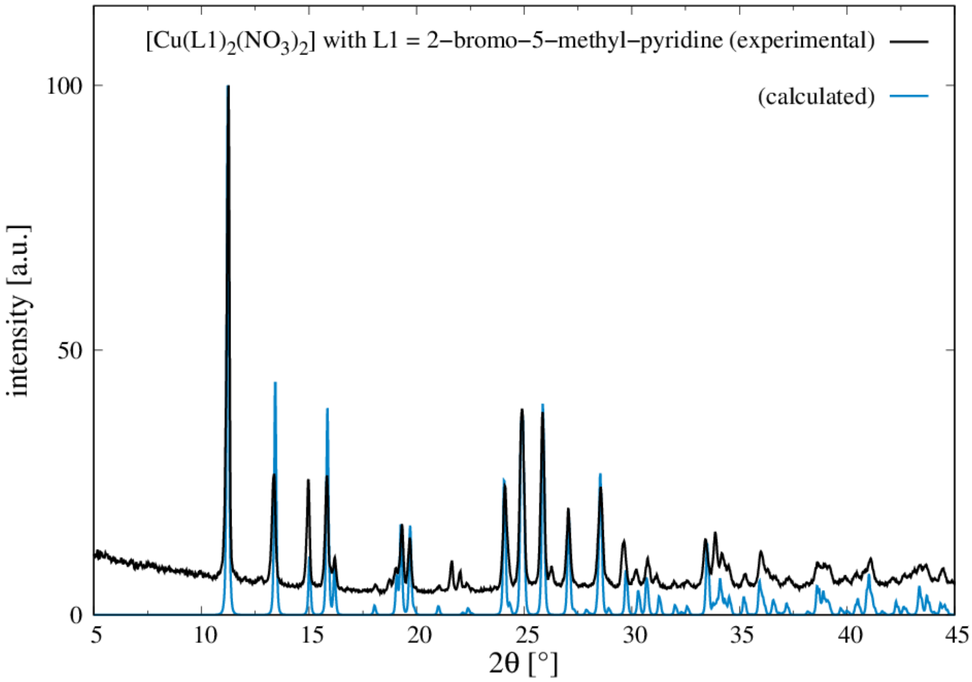

Figure S2. X-ray powder diffractogram of $[Cu(L^1)_2(NO_3)_2]$ (L^1 = 2-bromo-5-methylpyridine) measured with Cu-K$_\alpha$ radiation in reflection geometry (black) compared with a pattern simulated (blue) from the corresponding single crystal structure.

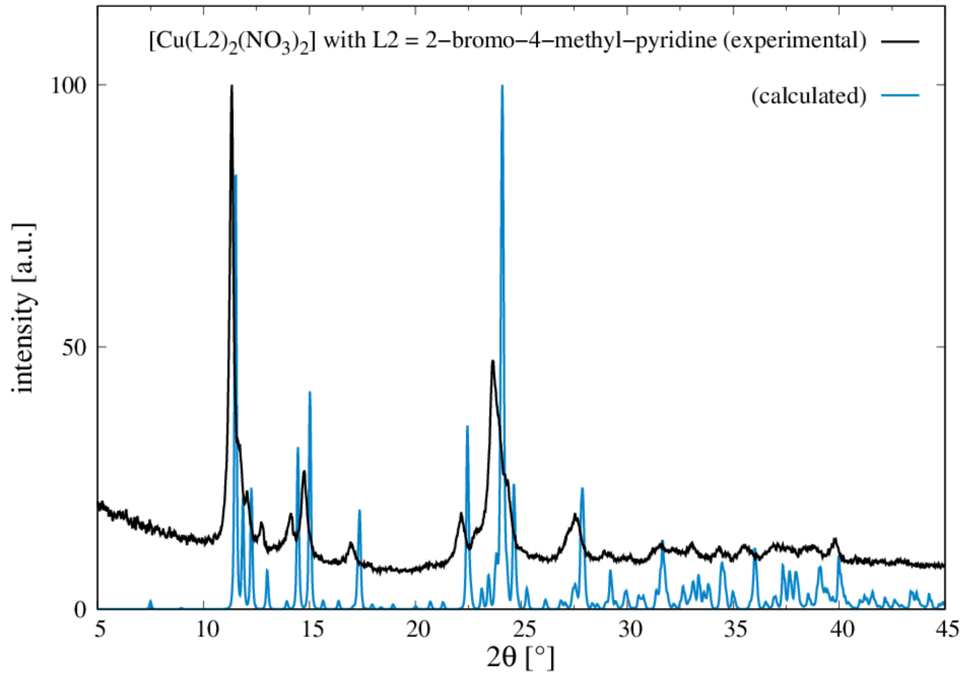

Figure S3. X-ray powder diffractogram of $[Cu(L^2)_2(NO_3)_2]$ (L^2 = 2-bromo-4-methylpyridine) measured with Cu-K_α radiation in reflection geometry (black) compared with a pattern simulated (blue) from the corresponding single crystal structure. Note: the positions of the Bragg reflections of the experimental and the simulated patterns differ due to different temperatures of the respective measurements (single-crystal data: 150(2) K; powder diffraction: 293(2) K).

$[Cu(L^1)_2(NO_3)_2]$	$[Cu(L^2)_2(NO_3)_2]$
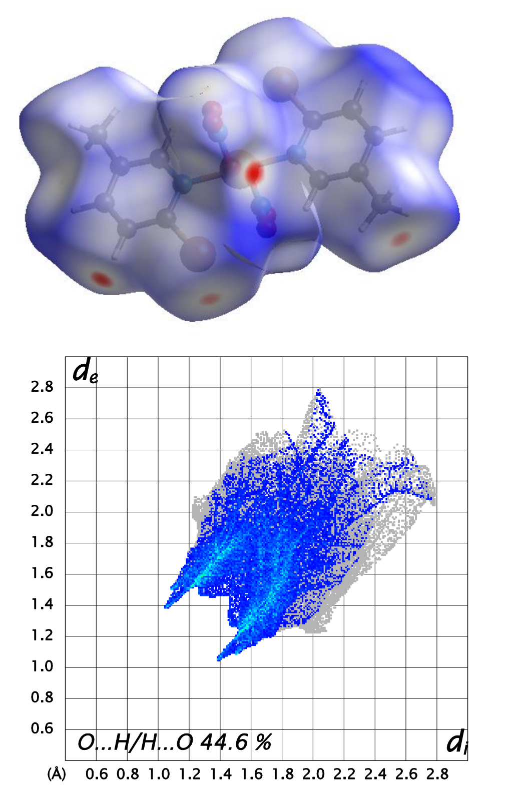	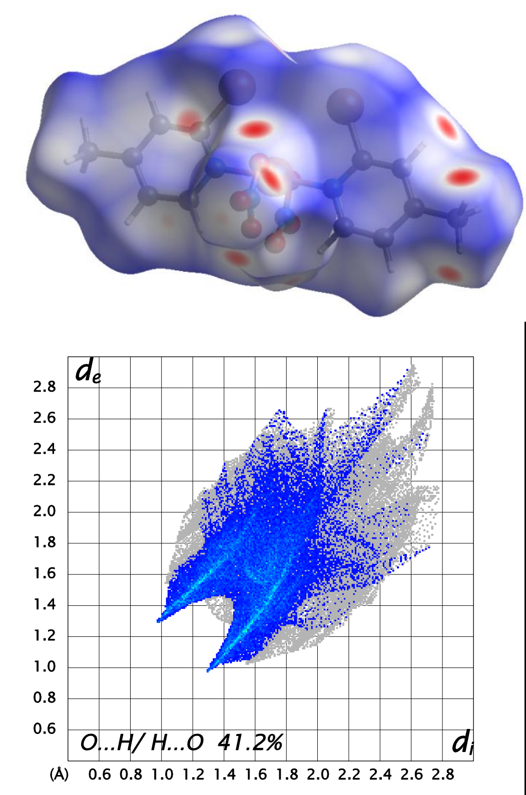

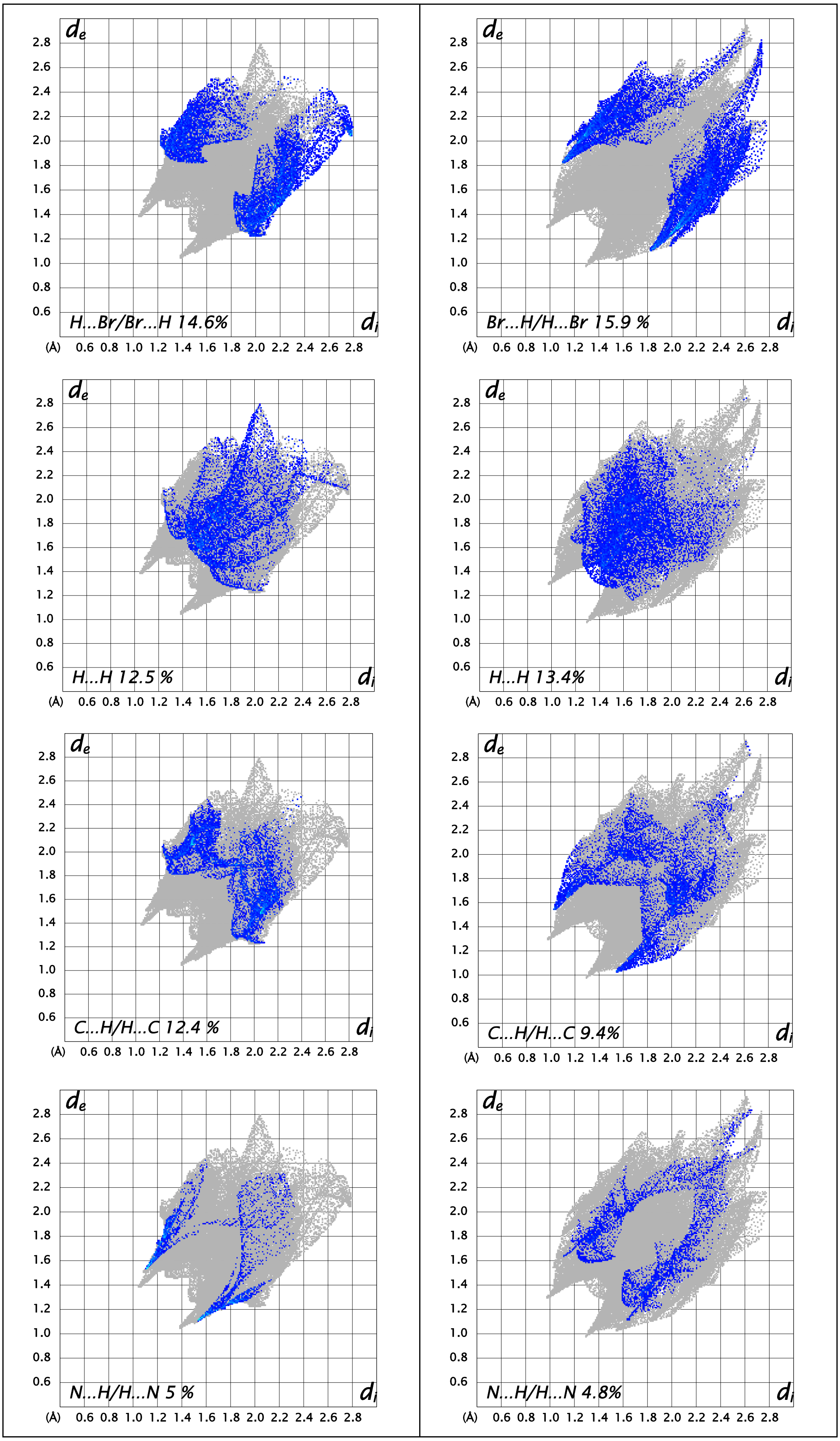

Figure S4. View of the Hirshfeld surfaces mapped over d_{norm} and Hirshfeld two-dimensional fingerprint plots for $[Cu(L^1)_2(NO_3)_2]$ and $[Cu(L^2)_2(NO_3)_2]$.

	$[Cu(L^1)_2(NO_3)_2]$ along *a*-axis	$[Cu(L^2)_2(NO_3)_2]$ along *b*-axis
**Coulomb energy**	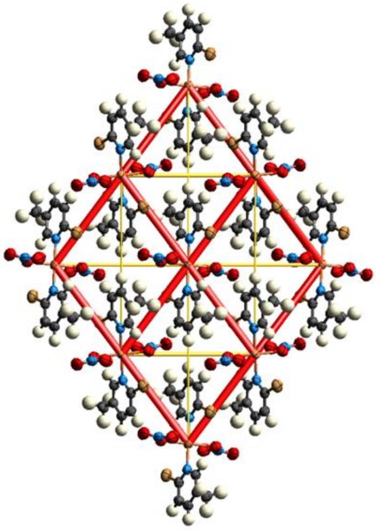	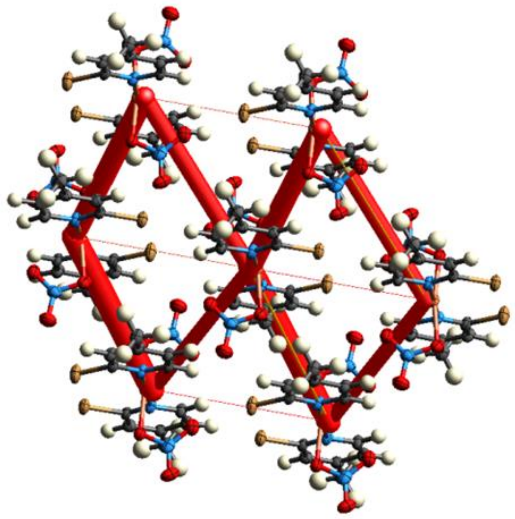
**Dispersion energy**	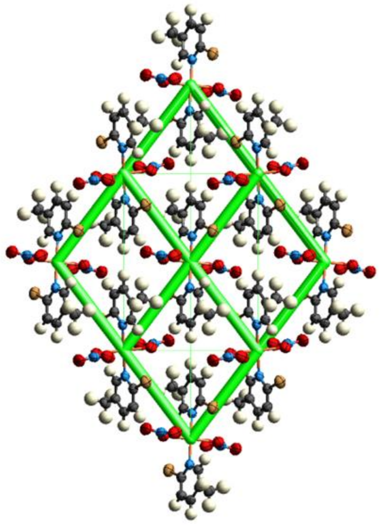	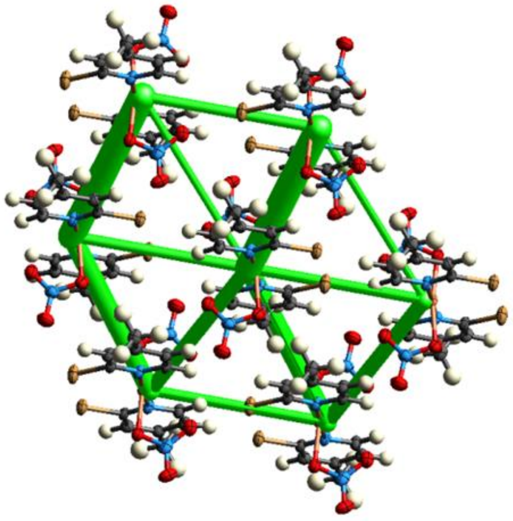
**Total energy**	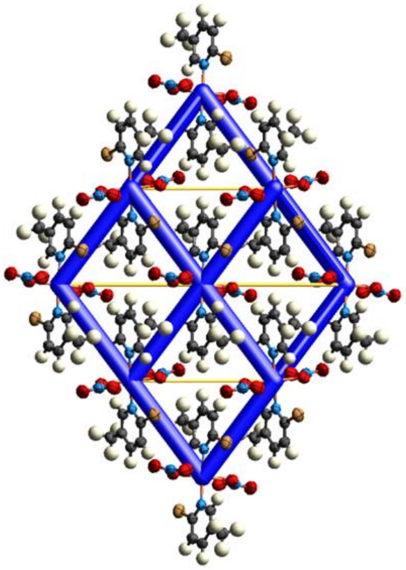	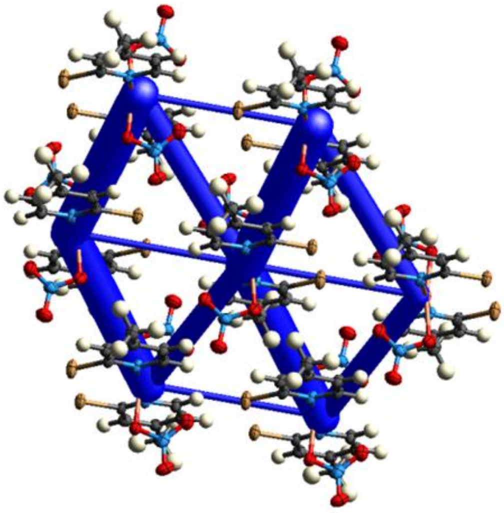

Figure S5. Energy frameworks constructed for Coulomb energy, dispersion energy and total energy for the both compounds $[Cu(L^1)_2(NO_3)_2]$ (L^1 = 2-bromo-5-methylpyridine) in $P2_1/c$ and $[Cu(L^2)_2(NO_3)_2]$ (L^2 = 2-bromo-4-methylpyridine) in $P\bar{1}$.

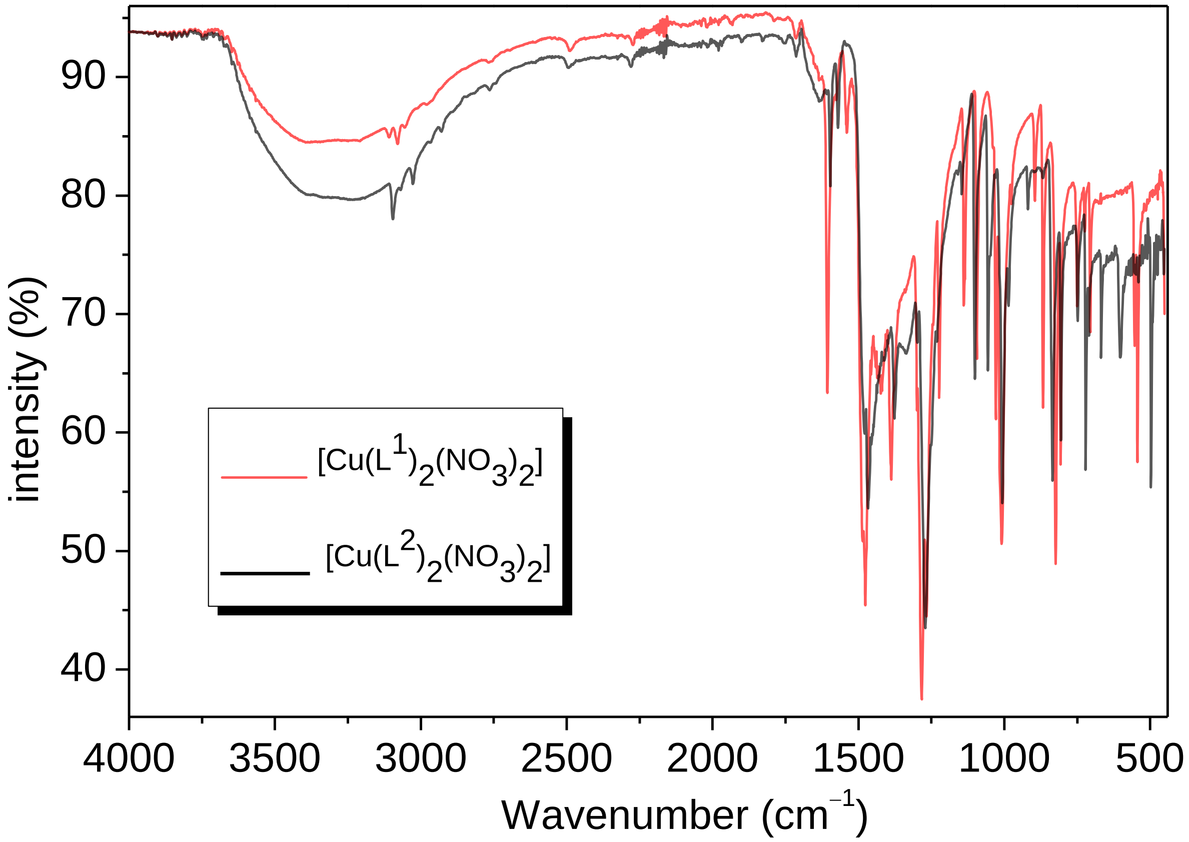

Figure S6. FT-IR spectra of the complexes $[Cu(L^1)_2(NO_3)_2]$ (L^1 = 2-bromo-5-methylpyridine) in $P2_1/c$ (red) and $[Cu(L^2)_2(NO_3)_2]$ (L^2 = 2-bromo-4-methylpyridine) in $P\bar{1}$ (black).

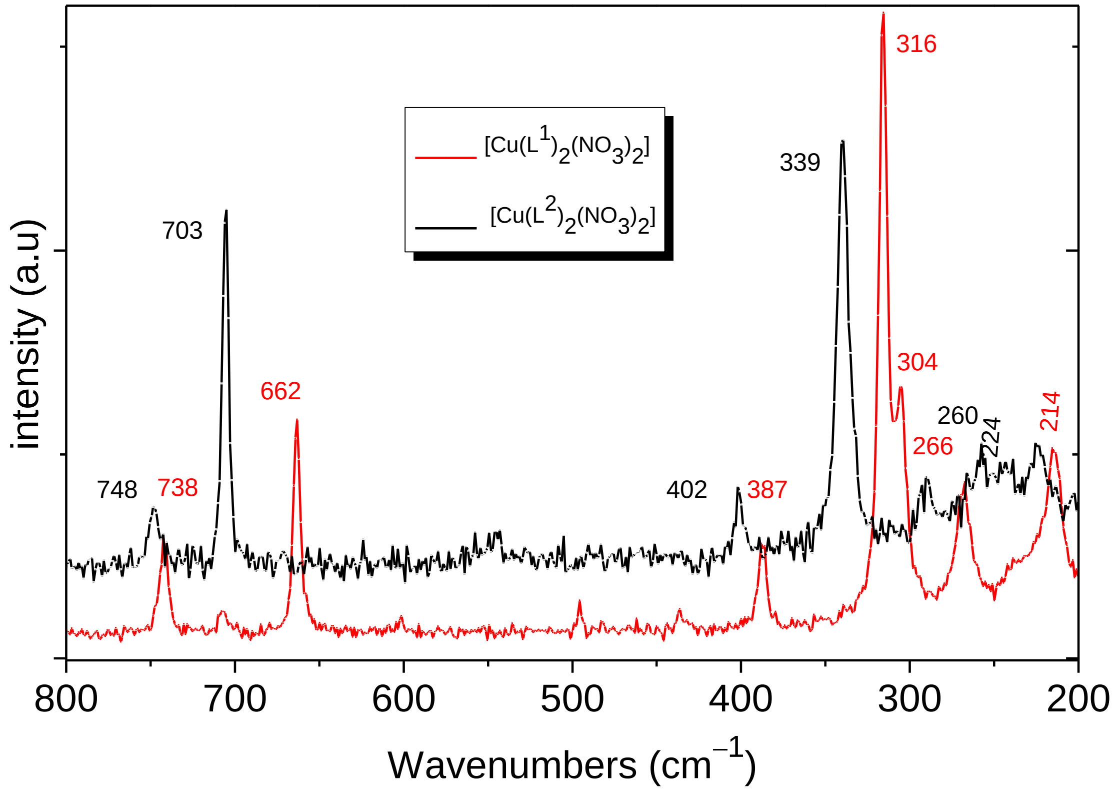

Figure S7. Raman spectra of the complexes $[Cu(L^1)_2(NO_3)_2]$ (L^1 = 2-bromo-5-methylpyridine) in $P2_1/c$ (red) and $[Cu(L^2)_2(NO_3)_2]$ (L^2 = 2-bromo-4-methylpyridine) in $P\bar{1}$ (black).

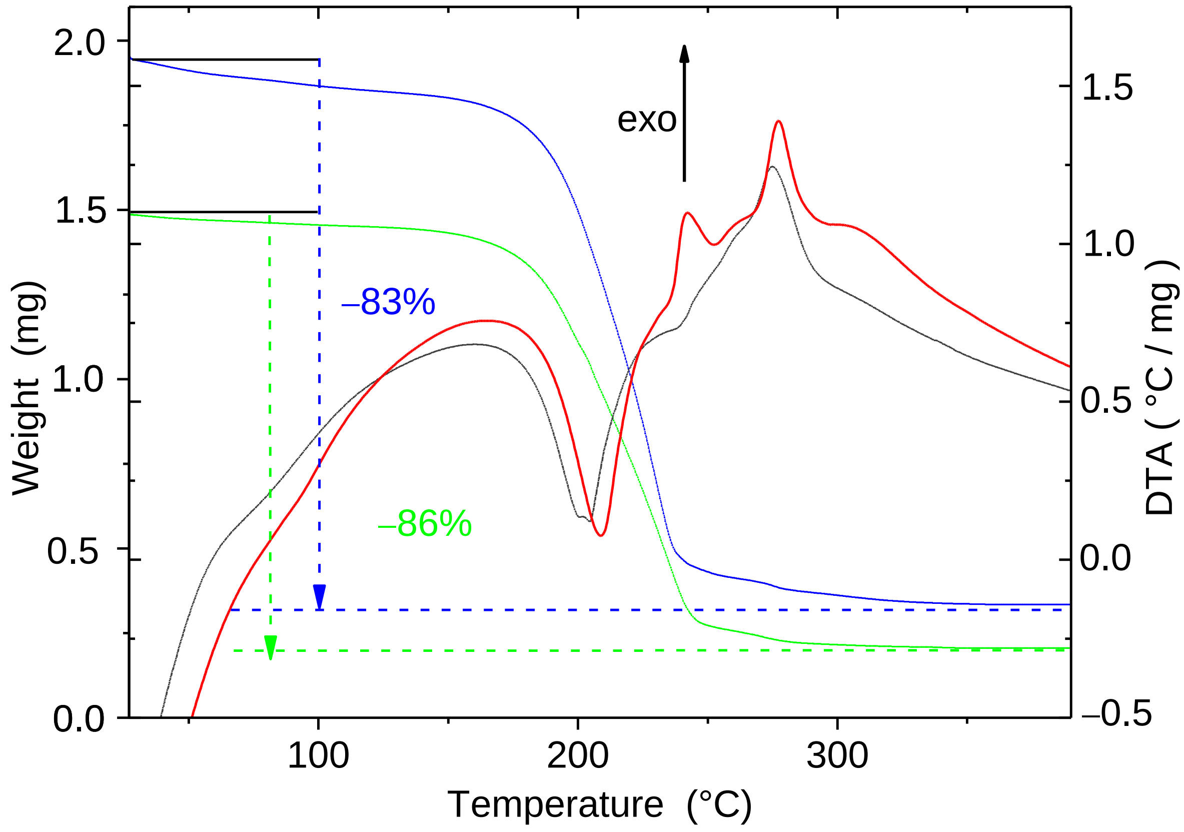

Figure S8. TG-DTA curves of $[Cu(L^1)_2(NO_3)_2]$ (blue and black lines) and $[Cu(L^2)_2(NO_3)_2]$ (green and red lines).

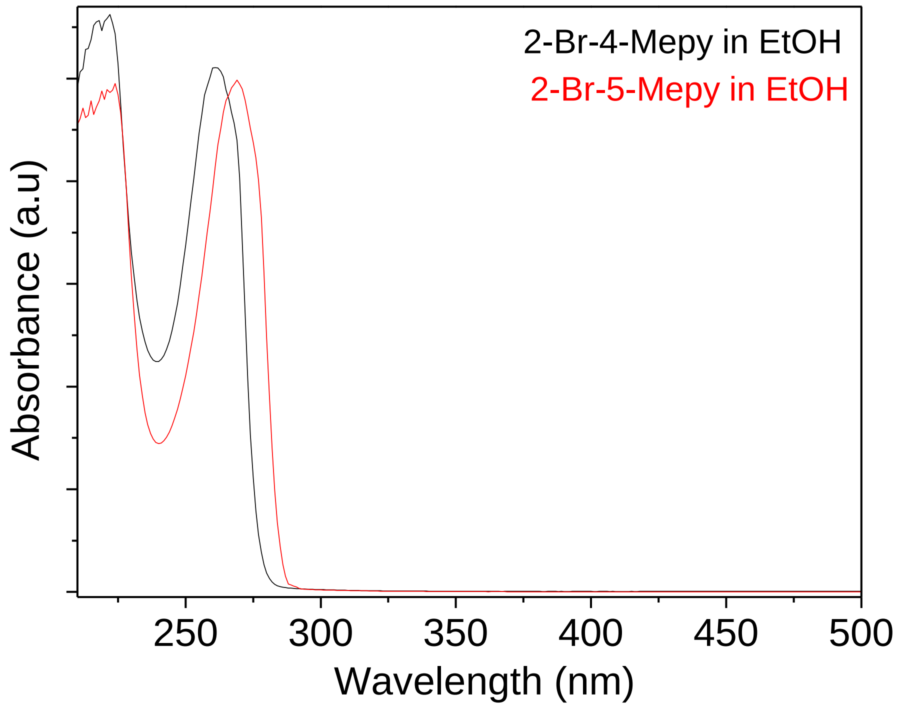

Figure S9. UV-vis absorption spectra of the ligands L^1 = 2-bromo-5-methylpyridine (red) and L^2 = 2-bromo-4-methylpyridine (black) in EtOH.

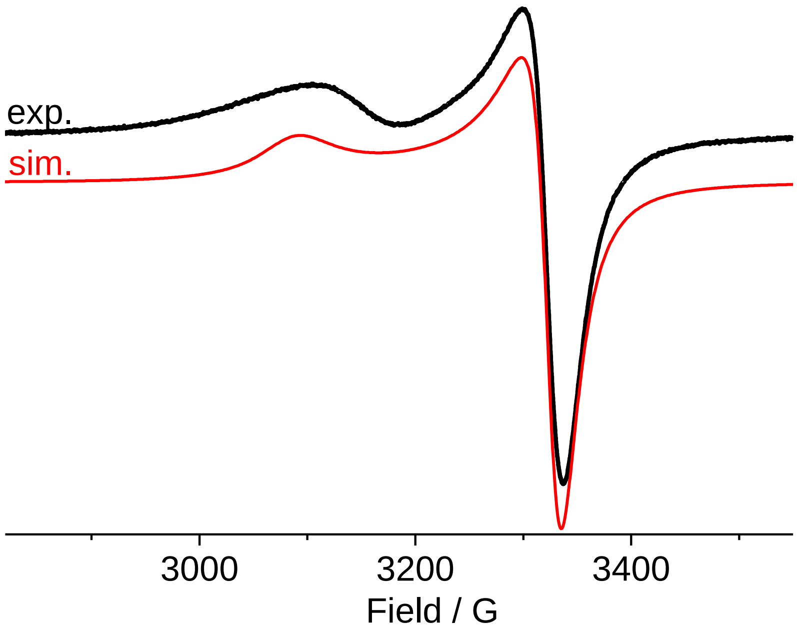

Figure S10. X-band EPR spectrum of $[Cu(L^1)_2(NO_3)_2]$, (L^1 = 2-bromo-5-methylpyridine) at 9.645555 GHz and 298 K. Receiver gain: 40, attenuation: 25 dB (0.3162 mW) (black), with simulation using $g_{||}$ = 2.230, $g_{\perp}$ = 2.070, and line width 50, 27 and 27 G (red). This makes g_{av} = 2.123; Δg = 0.160.

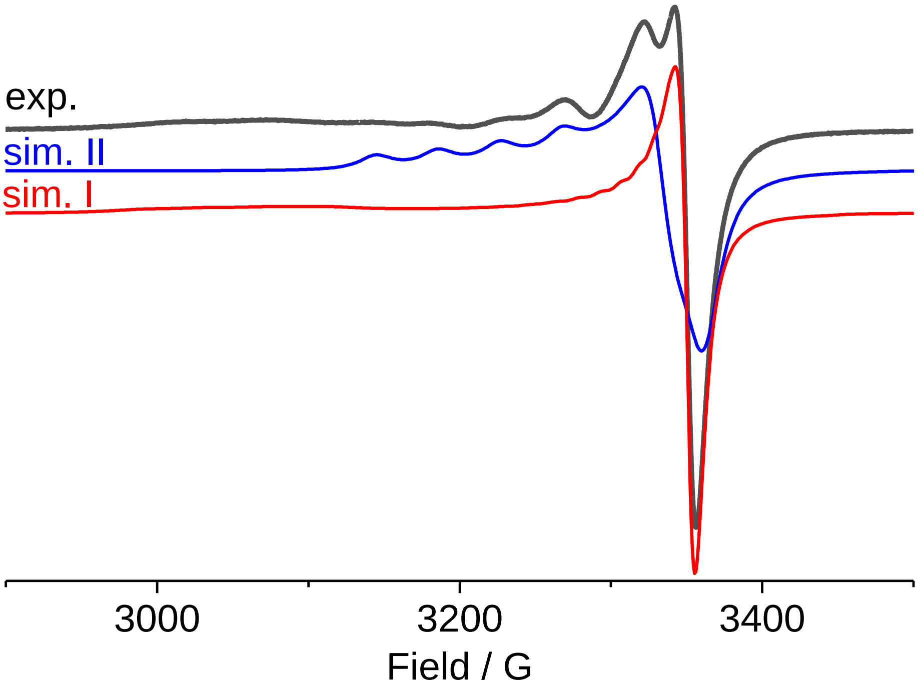

Figure S11. X-band EPR spectrum of $[Cu(L^2)_2(NO_3)_2]$, (L^2 = 2-bromo-4-methylpyridine) at 9.642502 GHz and 298 K. Receiver gain: 40, attenuation: 25 dB (0.3162 mW) (black), with simulation using $g_{||}$ = 2.260, $g_{\perp}$ = 2.057 and line width 95, 22 and 22 G (red). This makes g_{av} = 2.125; Δg = 0.203. A minor species is simulated using $g_{||}$ = 2.1352, $g_{\perp}$ = 2.057, $A_{||Cu}$ = 40 G, and line width 15, 10 and 10 Gauss. This makes g_{av} = 2.083; Δg = 0.078 (blue line).

Supplementary Tables

Table S1. Structure solution and refinement data for the two isomeric forms of $[Cu(L)_2(NO_3)_2]$

	L^1 = 2-bromo-5-methylpyridine	L^2 = 2-bromo-4-methylpyridine
Formula weight (g mol^{-1})	531.62	531.62
Crystalsystem	monoclinic	triclinic
Temperature (K)	293(2)	150(2)
Space group	$P2_1/c$	$P\bar{1}$
cell a (Å)	6.7738(7)	7.4916(5)
b (Å)	13.1890(9)	10.0404(7)
c (Å)	10.0999(10)	12.0723(8)
α (°)	90	86.176(6)
β (°)	103.298(8)	77.769(5)
γ (°)	90	79.793(5)
V (Å^3)	878.13(14)	873.00(10)
Z	2	2
Abs. coefficient (mm^{-1})	5.830	5.865
Density calcd. (g·cm^{-3})	2.011	2.022
F(000)	518	518
θ range (°)	2.584 to 29.354	1.727 to 29.142
Index range	$-9 \le h \le 9$<br>$-18 \le k \le 18$<br>$-13 \le l \le 13$	$-8 \le h \le 10$<br>$-13 \le k \le 13$<br>$-16 \le l \le 16$
completeness to θ = 25.35°	100%	98.8%
Reflections collected (R_{int})	16219 (0.102)	10362 (0.065)
Independent refl. (R_{sigma})	2401 (0.046)	4614 (0.065)
Goodness-of-fit on F^2	1.01	1.02
Weighting scheme ($P = (F_o^2+2F_c^2)/3$)	$w = [\sigma^2(F_o^2) + (0.1161P)^2]^{-1}$	$w = [\sigma^2(F_o^2)+(0.0532P)^2+0.6888P]^{-1}$
Extinct. coefficient	0.026(4)	-
Final R indices [I>2sigma(I)]	0.063	0.041
R indices (all data)	0.100	0.069
Largest diff. peak / hole (eÅ^{-3})	–0.78 / 0.69	–1.02 / 0.65
CCDC	2207776	2207769

Table S2. Selected metrical data of the isomeric forms of $[Cu(L)_2(NO_3)_2]$.

distances (Å)	L^1 = 2-bromo-5-methylpyridine	L^2 = 2-bromo-4-methylpyridine
Cu–O1	2.030(5)	1.967(2)
Cu–O1^i (L^1)	2.030(5)	
Cu–O6 (L^2)		2.4462(2)
Cu–O2	2.515(7)	2.445(3)
Cu–O2^i (L^1)	2.515(7)	
Cu–O4 (L^2)		1.967(3)
Cu–N1	1.990(5)	2.007(3)
Cu–N1^i(L^1)	1.990(5)	
Cu–N2(L^2)		2.013(3)
Cu···N3	2.673(6)	2.609(4)
Cu···Br1	3.328(9)	3.2614(2)
Cu···Br1^i(L^1)	3.328(9)	
Cu···Br2(L^2)		3.2621(7)

Br···Br		3.6929(8)
angles (°)		
N1–Cu–N1^i(L^1)	180.00	
N1–Cu–N2(L^2)		177.3(1)
N1–Cu–O2(L^1)	89.7(2)	
N1–Cu–O1(L^2)		90.4(1)
N1–Cu–O2^i(L^1)	88.0(2)	
N1–Cu–O4(L^2)		89.6(1)
O2–Cu–O2^i(L^1)	180.00	
O4–Cu–O1(L^2)		170.2(1)
Cu–O1–N3(L^1)	105.7(4)	
Cu–O1–N3(L^2)		102.7(2)
Cu–O1–N3^i(L^1)	152.7(4)	
Cu–O4–N4(L^2)		109.7(3)
CCDC	2207776	2207769

Symmetry code: (i) −x+2, −y, −z+2.

Table S3. Hydrogen bond details of the isomeric forms of $[Cu(L)_2(NO_3)_2]$[a].

L^1 = 2-bromo-5-methylpyridine in $P2_1/c$					L^2 = 2-bromo-4-methylpyridine in $P\bar{1}$				
D–H···A	$d_{D–H}$[b]	$d_{H···A}$	$d_{D···A}$	angle D–H···A	D–H···A	$d_{D–H}$[b]	$d_{H···A}$	$d_{D···A}$	angle D–H···A
C4–H4···O2	0.930	2.576	3.482	164	C4–H4···O3	0.930	2.586	3.513	174
C6–H6A···O3	0.960	2.580	3.477	155	C7–H7···O6	0.930	2.556	3.074	115
C6–H6C···Br1	0.960	2.930	3.740	143	C10–H10···O5	0.930	2.493	3.371	157
					C12–H12A···O6	0.960	2.480	3.298	143
					C12–H12C···O4	0.960	2.390	3.236	147

[a] Distances d (Å) and angles (°). [b] Distances fixed through AFIX instructions.

Table S4. Interaction energies (kJ/mol) of the molecular pairs calculated from energy framework calculation of $[Cu(L^1)_2(NO_3)_2]$ (top) and $[Cu(L^2)_2(NO_3)_2]$ (bottom).[a]

$[Cu(L^1)_2(NO_3)_2]$:

	N	Symmetry operation	R	Electron Density	E_{ele}	E_{pol}	E_{dis}	E_{rep}	E_{tot}
	1	x, y, z	6.77	HF/3-21G	–19.5	–12.6	–48.4	27.0	–49.7
	4	–x, y+1/2, –z+1/2	8.31	HF/3-21G	–29.3	–11.4	–47.1	22.9	–61.2
	2	–x, y+1/2, –z+1/2	9.96	HF/3-21G	–17.1	–5.1	–8.6	3.3	–25.8
	1	x, y, z	13.19	HF/3-21G	5.2	–1.3	–4.0	0.1	0.1
	2	–x, y+1/2, –z+1/2	11.43	HF/3-21G	0.5	–0.5	–8.4	5.6	–2.9
	0	x, y, z	10.79	HF/3-21G	14.5	–1.3	–1.8	0.0	12.4
	1	x, y, z	14.83	HF/3-21G	5.9	–0.8	–2.3	0.0	3.5

$[Cu(L^2)_2(NO_3)_2]$:

	N	Symmetry operation	R	Electron Density	E_{ele}	E_{pol}	E_{dis}	E_{rep}	E_{tot}
	1	x, y, z	10.04	HF/3-21G	–18.3	–7.8	–47.4	25.0	–46.2
	0	–x,–y,–z	9.50	HF/3-21G	–30.4	–11.5	–35.1	26.3	–48.8
	1	x, y, z	7.49	HF/3-21G	0.3	–5.0	–25.4	13.9	–14.7
	0	–x,–y,–z	7.58	HF/3-21G	–43.2	–17.8	–62.4	37.6	–81.3
	0	–x,–y,–z	9.87	HF/3-21G	–35.6	–10.2	–17.9	10.5	–50.5
	1	–x,–y,–z	8.68	HF/3-21G	–5.2	–9.6	–17.6	6.7	–22.0
	1	x, y, z	11.41	HF/3-21G	–1.3	–1.5	–8.7	2.1	–8.4
	1	–x,–y,–z	7.88	HF/3-21G	–48.3	–17.8	–45.4	32.9	–75.0
	1	–x,–y,–z	16.37	HF/3-21G	3.8	–0.6	–2.2	0.2	1.6

	0	–x,–y,–z	15.87	HF/3-21G	5.3	–0.9	–5.4	2.5	2.0
	1	–x,–y,–z	15.96	HF/3-21G	4.3	–0.4	–1.4	0.0	2.8

Energy model	K_{ele}	K_{pol}	K_{dis}	K_{rep}
CE–HF···HF/3-21G electron densities	1.019	0.651	0.901	0.811

[a] R is the distance between molecular centroids (mean atomic position) in Å.
